# Rising Global Temperatures and Kidney Health: A Comprehensive Review of Current Evidence

**DOI:** 10.3390/life15121897

**Published:** 2025-12-11

**Authors:** Evelina Valcheva, Nikolay Dimov

**Affiliations:** 1 Clinic of Nephrology, University Multiprofile Hospital for Active Treatment “Sv. Georgi”—Plovdiv, 15A Vasil Aprilov Blvd., 4002 Plovdiv, Bulgaria; nikolai.r.dimov@gmail.com; 2 Nephrology Section, Second Department of Internal Diseases, Medical University of Plovdiv, 15A Vasil Aprilov Blvd., 4002 Plovdiv, Bulgaria

**Keywords:** climate change, heat, kidney function, kidney diseases

## Abstract

Climate change significantly impacts natural environments, economies, and human health, with rising global temperatures causing increasingly frequent extreme weather events. Studies have shown that heat exposure accelerates kidney function decline and increases hospitalizations for renal diseases. Heat contributes to kidney disease through mechanisms such as dehydration, ischemic injury, inflammation, and oxidative stress, leading to acute kidney injury, chronic kidney disease, increased risk of kidney stone formation, and proteinuria. In light of the anticipated rise in temperatures in the forthcoming years, it is of crucial importance to know the possible consequences for kidney health. This review highlights the relationship between these phenomena, the potential complications that may arise, and the mechanisms of their occurrence. The identification of these mechanisms will further support the development of future targeted preventive strategies and interventions.

## 1. Introduction

Climate change and, in particular, global warming constitute one of the major challenges that humanity faces in the 21st century, already causing significant shifts in global weather patterns and an increase in extreme weather events. Climate change modifies weather systems by raising global temperatures, altering atmospheric circulation, and changing precipitation patterns [1]. These climatic shifts pose significant threats to ecosystems, human health, agriculture, water resources, and infrastructure [2]. Global temperatures are rising, weather conditions are becoming more erratic, and extreme weather phenomena such as longer-lasting heatwaves, wildfires, and droughts are occurring more often. As a result, vulnerable populations, especially in developing countries, are disproportionately affected both economically and health-wise, facing increased risks of malnutrition, heat stress, vector-borne diseases, and other climate-related impacts [3].

Extreme heat plays a significant role in climate change, as global warming results in more frequent, severe, and extended heatwaves. Heat increasingly endangers public health and safety by causing direct heat-related illnesses [4]. Extreme heat events or heat waves, defined as a period where local excess heat persists over a sequence of consecutive sweltering days and nights, have spread rapidly into new regions of the globe at unseasonable times of the year and represent a major public health challenge [5]. The severity of this problem is expected to increase in the coming years.

Worldwide temperatures are increasing rapidly at a rate of about 0.27 °C every ten years, far exceeding any warming rates recorded in the geological past [6]. In just the past two years, the global temperature has surged by over 0.4 °C, reaching a 12-month peak in August 2024 that was about 1.6 °C above the temperatures recorded at the start of the 20th century [7]. Climate models suggest that global surface temperature could rise between 1.5 °C and 5.5 °C compared with the pre-industrial period by 2100 [8].

The direct and observable health consequences of severe climate-related events, including increased illness and death, are more frequently being attributed to climate change.

Multiple studies have demonstrated that sudden temperature fluctuations, such as cold spells or heat waves, directly influence hospital admission rates, illness, and death. In New York during a five-year period (2018–2022), an estimated 525 people died annually due to heat, accounting for about 3% of all deaths over the warm season (May through September) [9]. Heat-waves cause excess deaths, with heat ranking among the top weather-related causes of mortality in high-income countries [10]. Research shows that globally an average of approximately 489,000 heat-related deaths occurred annually between 2000 and 2019, and by mid-century, heat could cause an additional 250,000 deaths per year [4]. Heat exposure significantly contributes to more fatalities from cardiovascular pulmonary and renal diseases, can worsen mental health and negatively impacts pregnancy outcomes, making it the deadliest weather phenomenon.

Heat affects the body by raising the core temperature, which is regulated through sweating and blood flow to the skin. During extreme heat exposure, these mechanisms can become overwhelmed, causing heat-related illness. Heat exhaustion symptoms include dizziness, headaches, muscle cramps, and heavy sweating due to dehydration and salt loss. If untreated, it can progress to heat stroke, where the body temperature exceeds 40 °C (104 °F), cooling mechanisms fail, and neurological symptoms occur.

The consequences of climate change are especially harmful to kidney health, as environmental issues significantly worsen kidney function [11]. Certain groups, including elderly individuals, children, outdoor workers, and those lacking access to cooling systems, are particularly susceptible to heat-related health hazards [12].

Future preventative strategies should focus on characterizing heat risks in vulnerable populations, developing organ-protective interventions, assessing drug impacts on thermal regulation, determining human thermal tolerance limits, and studying the effects of physical activity and nutrition on heat tolerance [13]. Protocols based on the main adaptation strategies (behavioral, physiological, and societal/structural) must be developed to translate research into occupational and community strategies [13].

Kidney injury may manifest in multiple forms, with one major outcome being dehydration and loss of body fluids [14]. Heat exposure and volume depletion are known risk factors for nephrolithiasis and acute kidney injury (AKI). Heat stress is also increasingly recognized as a key contributor to CKD of unknown origin (CKDu), particularly in tropical regions with consistently high temperatures [15]. Additionally, heat contributes to proteinuria primarily by inducing kidney stress due to dehydration, which raises glomerular permeability and impairs renal tubular function, potentially resulting in chronic proteinuric kidney disease with prolonged exposure [16].

This review of literature presents the role of heat stress, its diverse impact on kidney function and, respectively, the occurrence of kidney diseases closely associated with heat exposure.

There are multiple patterns of renal injury, and in the following paragraphs, the most common forms of kidney injury associated with heat exposure will be presented in detailed manner.

## 2. Heat Stress and Renal Involvement

### 2.1. Heat Stress and Proteinuria

The first published reports linking kidney function and environmental temperature date back to 1898 in a report by Sollmann and McComb [17]. Their initial observations documented that urinary protein content varied inversely with cool ambient temperatures [17]. However, high temperatures also can lead to proteinuria. Sato et al. conducted an experimental study with mice that were exposed to heat [18]. Core temperature remained unchanged in the control group. Conversely, the temperature of heat-exposed mice reached 40.92 °C. The authors found a statistically significant correlation between the higher core body temperatures and the level of albuminuria [18]. In another study, Kwag et al. demonstrated that the number of patients showing proteinuria symptoms during health check-ups is significantly elevated as the number of heat wave days increased [19].

Recurrent episodes of heat stress combined with dehydration cause kidney vasoconstriction and trigger hormone release, including vasopressin, which increases urinary concentration and leads to elevated tubular workload [15]. Heat stress also triggers the release of inflammatory mediators, including cytokines and chemokines, which contribute to tubulointerstitial inflammation and injury, further compromising glomerular filtration barrier integrity and promoting proteinuria [20].

Exposure to environmental stress can strengthen local tissue resilience, representing a form of kidney self-defense. Thermotolerance serves as an example of such adaptive defense. The exposure of tissues or cultured cells to heat stress triggers the production of stress proteins known as heat shock proteins (HSPs)—one of the most effective known kidney protection mechanisms [21]. These HSPs, functioning as molecular chaperones, play a key role in enhancing kidney protection against subsequent harmful stimuli [21]. Heat shock proteins (HSPs), particularly HSP70 and HSP90, play a cytoprotective role by mitigating cellular damage, protein aggregation, and apoptosis in renal cells under heat stress, but dysregulation or overwhelming heat injury can impair this protective effect and contribute to the kidney injury progression [16].

### 2.2. Heat Stress and Hematuria

**Figure 1:**
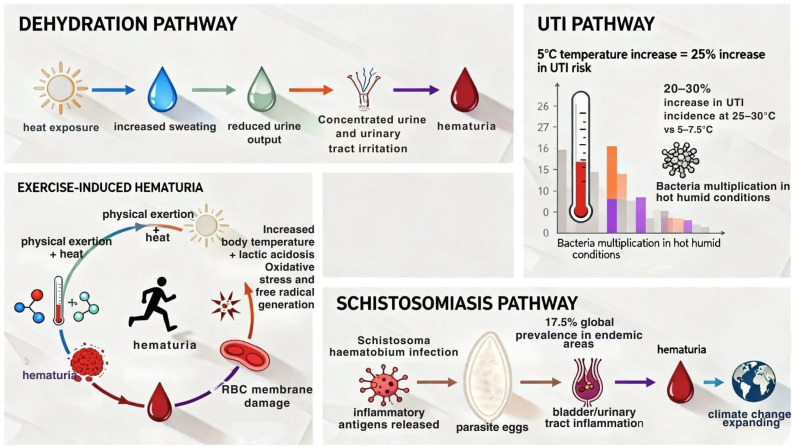
The heat exposure pathways leading to hematuria. Abbreviations: UTI, urinary tract infection.

A variety of conditions exacerbated by heat, can lead to hematuria (Figure 1).

Heat exposure, especially in summer, increases sweating and reduces urine output, causing dehydration, which in turn may cause concentrated urine and irritation of the urinary tract, leading to microscopic or gross hematuria.

UTIs become more common in hot, humid conditions and can clinically present with hematuria [22]. Large-scale studies analyzing over 15 million UTI cases across 400 metropolitan areas in the United States (US) demonstrate a clear temperature-UTI association [22]. A temperature increase of 5 °C is associated with a 25% increase in UTI risk. UTI incidence increased by 20–30% in temperature around 25–30 °C compared to cooler temperatures of 5–7.5 °C [22].

Exercise-induced hematuria, is another cause where physical exertion combined with heat leads to transient blood in urine [23]. Increased body temperature and lactic acidosis during strenuous exercise contribute to oxidative stress and free radical generation, leading to red blood cell membrane damage [24].

Lastly, infection with Schistosoma haematobium, the parasite that causes urogenital schistosomiasis, often presents with hematuria. Hematuria results from inflammation of the bladder and urinary tract caused by the release of inflammatory antigens by parasite’s eggs [25]. A systematic review and meta-analysis covering women between 2016 and 2020 found a global weighted prevalence of about 17.5% for female urinary schistosomiasis in endemic areas of Africa and the Middle East [26]. Climate change is anticipated to shift endemic zones and transmission intensity of Schistosoma haematobium. Rising temperatures due to climate change may expand or shift schistosomiasis-suitable regions where mean annual temperatures are below the thermal optimum, thereby potentially increasing prevalence in those area [27]. Meanwhile, the ecology of the intermediate snail host, Bulinus globosus, is significantly affected, which is critical for the parasite’s lifecycle [28].

### 2.3. Heat Stress and Nephrolithiasis

It is estimated that rising greenhouse gas emissions and overall pollution will lead to an increase in the average global temperature by 1.5–4.5 °C [29]. Ongoing climate change and global warming are anticipated to impact certain health conditions that are sensitive to temperature changes, such as nephrolithiasis. Heat causes increased transdermal fluid loss, leading to elevated serum osmolality and increased vasopressin secretion by the posterior pituitary. This results in increased urinary concentration and reduced urinary volume, raising the concentration of relatively insoluble salts such as calcium oxalate above their solubility threshold, promoting crystallization and stone formation. Low humidity produces a similar effect through increased insensible water losses. Heat also increases vitamin D activation, which raises urinary calcium levels, and causes electrolyte imbalances, further enhancing stone formation [30]. Behavioral factors like limiting fluid intake may further increase stone risk.

A landmark study by Brikowski et al. used a climate model with moderate warming, and found that the proportion of Americans living in a “high risk” zone for kidney stones is projected to rise from 40% in 2000 to 56% by 2050, and could reach 70% by 2095 due to climate change and rising temperatures [31]. It was estimated that, there would be a climate-related rise of 1.6–2.2 million lifetime cases of nephrolithiasis by 2050 [31]. More recently made models in South Carolina suggests that, under moderate climate change, there will be about 5938 additional emergent kidney stone cases attributable to heat between 2025 and 2089, while under more severe climate change, the increase could be 10,431 cases [32].

Other studies conducted all around the world demonstrate the unequivocal link between heat and nephrolithiasis. Epidemiological studies show that kidney stone incidence spikes sharply after hot weather events in hot regions [31]. In Iran, a positive correlation between stone prevalence and both temperature and sunlight index was found for both men and women [33]. Studies in Japan found higher incidence rates in the summer months and lower incidence rates in the winter months in both Tokyo and in a rural area [34,35]. In Saudi Arabia, a country with a desert climate and an extremely hot summer, in a 3-year study of emergency room visits, regression analysis found a statistically significant correlation between the mean monthly temperature and the number of males presenting with urinary colic [36]. Abreu-Jr and Ferreira Filho published a study examining the relationship between ambient temperature, humidity, and hospitalizations for nephrolithiasis across different climate zones in Brazil [37]. The research found that for each 1 °C increase in monthly mean temperature, there were approximately 500 additional hospitalizations per month for nephrolithiasis, translating to more than 7000 new kidney stone hospitalizations annually per 1 °C temperature increase [37]. They also suggested that dry conditions exacerbate stone risk [37].

In the US, most research has been conducted based on the concept of the “stone belt,” a region, mainly in the southeastern states, known for its significantly higher prevalence of kidney stones. The Cancer Prevention Study II established the “stone belt” concept by mapping the prevalence of urolithiasis across the US, showing an increased prevalence from north to south and west to east [38]. Researchers linked this tendency to elevated temperature and stone disease risk, attributing it to increased water loss through perspiration, leading to dehydration and higher urine concentration [39]. This has served as a key argument supporting the role of climate and heat exposure in the increased incidence of nephrolithiasis. On a global scale, rising temperatures are expected to escalate the prevalence of kidney stones, resulting in substantial increases in healthcare costs and wider socioeconomic impacts.

### 2.4. Heat Stress and AKI

In 2015, Wen et al. conducted the largest study globally and the first in Brazil to assess the relationship between ambient temperature and hospitalizations for renal diseases [40]. Covering 2,726,886 hospitalizations from 1816 cities between 2000 and 2015, the study used a time-stratified case-crossover design and found a positive association between daily mean temperature and the risk of renal disease hospitalizations [40].

Studies show that the risk of AKI episodes rises markedly on hot days, with one finding indicating a 62.3% increased odds of AKI on days when temperatures reach 32 °C compared to 17 °C [41]. The risk begins to increase with every 1 °C rise above 17 °C, and heatwaves have been linked to substantial spikes in AKI cases-up to a 28.6% increase during a 7-day heatwave [41]. The most pronounced effects occur the day before the AKI episode is recorded [41].

**Figure 2:**
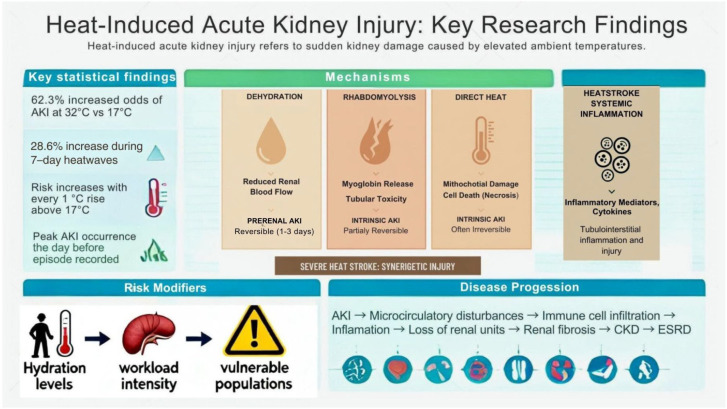
Mechanisms linked to heat exposure and AKI.

Heat exposure has a proven causal link to kidney disease through several mechanisms (Figure 2). Firstly, repeated or prolonged exposure to high temperatures leads to dehydration, which reduces kidney blood flow and causes ischemic injury [42]. Heat-induced rhabdomyolysis is another significant contributor to kidney injury, leading to electrolyte disturbances, particularly in conditions of exertional heat stroke, where intense physical exertion in hot environments causes muscle breakdown [20]. This leads to the release of myoglobin into the bloodstream, which causes AKI due to direct tubular toxicity, ischemia, and oxidative damage [43].

Extreme heat harms renal tubular epithelial cells, particularly in the proximal tubules. This damage occurs when heat-damaged mitochondria disrupt ATP production, thereby undermining cell viability [44]. Heat exposure disrupts mitochondrial energetics and fatty acid oxidation in renal cells, causing an imbalance in oxidative stress [44]. This activates the NLRP3 (NOD-, LRR- and pyrin domain-containing protein 3) inflammasome and releases proinflammatory cytokines that additionally aggravate the kidney dysfunction [45]. Heatstroke activates innate immunity, engaging neutrophils that generate extracellular traps (NETs) and NK/NKT cells that release inflammatory mediators [44]. These factors increase tubular necrosis and renal injury [44,45].

Heat stroke (HS) is a severe form of heat injury caused by either prolonged exposure to high temperature or intense physical exertion [46]. It is typically categorized into two types based on the presence of strenuous activity. Exertional heat stroke (EHS) occurs in otherwise healthy individuals, such as athletes or military personnel, following vigorous physical activity [47]. In contrast, classical heat stroke (CHS) primarily affects vulnerable populations, including the elderly or those with preexisting conditions [47].

In 2013, Naushad et al. studied ten individuals completing downhill runs causing muscle damage and flat runs without damage, followed by running in the heat (33 °C) [48]. Muscle damage elevated creatine kinase and interleukin-6 levels, increased kidney injury biomarkers such as NGAL, and reduced kidney function. Half of the participants met the AKIN criteria for AKI [48]. These findings suggest that muscle damage amplifies kidney stress during heat exposure in the presence of strenuous physical activity, highlighting it as a risk factor for AKI [48]. In another study by Schlader et al., 29 adults wearing firefighter gear performed uphill treadmill walking in a hot, humid chamber during short and long trials [49]. The longer trial resulted in greater increases in serum creatinine, NGAL, core temperature, and body fluid loss and decreased plasma volume. They concluded that AKI biomarkers are influenced by exercise-induced hyperthermia and hypovolemia [49].

Heat stress likely acts as a catalyst by causing repeated increases in core body temperature during physically demanding work in high heat, which may accelerate pre-existing kidney pathology or increase susceptibility to nephrotoxic injury, including from environmental or chemical toxins [50]. The impact on kidneys can range from low-grade damage detected by kidney injury markers to overt AKI, the latter commonly diagnosed after exertional heat stroke and identified through increased serum creatinine or reduced urine output [20]. Severe kidney injury often results in poor recovery and is linked to microcirculatory disturbances, immune cell infiltration, loss of functioning renal units, and renal fibrosis. As a result, AKI frequently progresses to CKD and eventually even to end-stage renal disease (ESRD) [51].

Clinical studies of patients with EHS have also documented hypophosphatemia (98%), hypocalcemia (70%), hyponatremia (34%), hypokalemia (32%), and hypomagnesemia (30%) as common electrolyte abnormalities [52]. The mechanism of hyponatremia in heat stroke differs from that of exercise-associated hyponatremia. In classic heat stroke, hypoelectrolytemia with tubular dysfunction develops as part of the acute physiological changes associated with hyperthermia, direct cytotoxicity of heat, and inflammatory and coagulation responses, resulting in injury to the vascular endothelium and kidney tissues [52].

HS-induced AKI is influenced by a diverse array of risk factors shaped by physiological, environmental, and pathological conditions. Heat exposure and strenuous physical exertion in high-temperature environments serve as primary triggers, particularly for individuals in labor-intensive occupations, such as construction workers and agricultural workers [46]. Studies shows that agricultural workers exposed to occupational heat stress have a notably high incidence of AKI even after just one work shift [46]. This risk is influenced by factors such as hydration levels, workload intensity, and payment systems like piece-rate compensation that may incentivize working without adequate rest. In a study by Chicas et al., epidemiological data show heat-exposed agricultural workers showed significant changes in metabolites and metabolic pathways compared to non-heat-exposed non-agricultural workers [53]. These changes include molecular signatures of impaired gluconeogenesis from recurrent proximal tubule injury, dysregulation of histidine metabolism and nitrogen elimination, and altered metabolic activity of commensal microorganisms [53]. Specifically, this study validated metabolites within the nitrogen elimination pathway, including arginine, citrulline, and uracil, which are associated with renal dysfunction, impaired renal metabolism, oxidative stress, and inflammation [53]. Therefore, repeated heat exposure in agricultural environments may affect these pathways, contributing to kidney dysfunction [53]. Furthermore, these changes can increase the risk of CKD development. The bidirectional link between AKI and CKD is well recognized, with a rise in incidence of AKI therefore potentially able to increase the incidence and progression of CKD [54].

### 2.5. Heat Stress and CKD

Climate change is exacerbating kidney disease risk by increasing both chronic heat exposure through gradually rising high temperatures and acute exposures through intensifying heat waves. An incremental increase in global surface temperature due to climate change, for example, 1 °C to 2 °C is likely to aggravate the risk of CKD [55].

Non-optimal temperatures contribute unevenly to the global CKD burden, which is influenced by socioeconomic and demographic inequalities. In lower-SDI settings, populations are more susceptible to heat, with high temperatures associated with higher CKD mortality and disability rates [56]. In high-SDI settings, low temperatures account for more of the CKD burden, although these regions show the fastest growth in heat-related CKD disability rates. Geographic contrasts are prominent, with hot regions such as North Africa and the Middle East carrying a high heat-related CKD burden, whereas certain colder countries show elevated CKD rates due to low temperatures [56]. Between 1990 and 2021, the equity patterns for heat- and cold-related CKD diverged. Heat-related socioeconomic disparities have worsened, with the CKD burden concentrated in less-advantaged populations. For cold-related CKD, the burden was concentrated in higher-SDI regions, suggesting narrowing socioeconomic gaps for cold exposure, whereas heat exposure gaps widened [56].

Extreme heat has been linked to both the development of CKD and the progression to ESKD. The term chronic kidney disease of unknown aetiology (CKDu) refers to CKD in the absence of diabetes, long-standing hypertension, glomerulonephritis, obstructive uropathy or other apparent causes, and is prevalent in certain rural populations around the world [57]. The earliest report resembling what is now known as CKDu was by Mani in 1993 [58]. He identified chronic interstitial nephritis as the primary cause of chronic renal failure among patients at a hospital in Chennai [58]. Similarly, Mesoamerican endemic nephropathy (MeN) is a form of CKDu, found in specific high-prevalence areas along the Pacific coast of the Mesoamerican region, extending from southwest Mexico to Costa Rica [59]. An epidemic of MeN was first reported in 2002 among sugarcane workers in El Salvador [60]. The occurrence is higher in several CKD hotspots worldwide, including regions in Sri Lanka, the Indian state of Andhra Pradesh, Pakistan, Egypt, and the coastal areas of Nicaragua, El Salvador, and Costa Rica [61]. Although the exact causal links between environmental and occupational factors and CKDu remain unclear, studying this condition has been recognized by the International Society of Nephrology as a global research priority [62].

There are different theories regarding the possible etiology of CKDu; however, the most widely accepted is the heat stress nephropathy hypothesis. While meta-analytic findings did not indicate a statistically significant association between temperature and CKD/CKDu morbidity, narrative evidence suggests a likely association in specific subpopulations, including males, agricultural workers under 50 years, those aged 65 and older, individuals of low socioeconomic status, and residents of rural, tropical, and subtropical regions [63].

CKDu is believed to result primarily from kidney injury caused by physical labor in hot environments [64]. Various studies shows this damage is predominantly tubular, with biomarkers such as urinary NGAL and IGFBP7 rising after heat-exhausting work [53]. These findings are consistent with renal biopsies from at-risk workers in Nicaragua that correspondingly reveal acute tubular injury and chronic tubulointerstitial nephritis [62]. Factors worsening injury include longer work hours, severity of hyperthermia, dehydration, muscle damage, and intake of sugary beverages rich in fructose [64]. Heat stress reduces kidney blood flow, causing local hypoxia and ATP depletion, which triggers inflammation, oxidative stress, and tubular damage [20]. Animal studies support these findings, showing that repeated heat exposure without hydration leads to kidney injury, while access to water offers protection [65]. This framework links heat stress, dehydration, and metabolic strain to the development and progression of CKDnt.

The disease predominantly affects men engaged in physically demanding outdoor occupations like agriculture (corn and rice farmers, cotton plantation workers), construction and mining. CKDu cause sustained decreases in GFR that often occur without the presence of traditional kidney disease risk factors (older age, hypertension, diabetes and obesity) with absent or minimal proteinuria and is occasionally accompanied by microhematuria [59,60]. In its advanced stages clinically, it may present with progressive kidney failure, hyperuricemia, hypokalaemia, hyponatraemia, hypertension and mild anaemia [53]. The diagnosis is supported by ultrasound showing normal-sized kidneys with increased echogenicity and the absence of obstruction [66]. When performed, a kidney biopsy shows chronic tubulointerstitial disease, usually along with glomerulosclerosis and signs of kidney ischemia but only mild vascular lesions [67,68]. Progression to ESRD occurs over several years and is higher in those who work more harvests. Kidney replacement therapy is rarely available in the affected regions, and many thousands have died as a result [66]. Research into the causes of chronic kidney disease of unknown origin (CKDu) remains ongoing, with no definitive cause identified yet. Due to inconsistent case definitions and limited high-quality data, understanding CKDu’s global burden and causes remains incomplete. Further studies are crucial to clarify its etiology and to develop effective prevention and treatment strategies.

Another potentially dangerous but hidden link between heat and kidney health, particularly chronic kidney damage, is that low birth weight (LBW) is closely associated with a higher risk of CKD. Studies, including an analysis of 126,000 women in India and Pakistan, found that heat exposure in the second trimester increases the risks of premature birth (PTB) and LBW [69]. A meta-analysis showed a 9% higher LBW risk and a 16% higher PTB risk during hotter temperatures [70]. Brenner et al. linked LBW to a reduced nephron number, increased hypertension, and CKD risk [71]. This developmental programming hypothesis is supported by studies showing that LBW is a marker of a lower nephron number and increased disease risk [72].

Potential heat-related complications such as dehydration and rhabdomyolysis can also lead to CKD in several ways. As previously mentioned, these complications may cause AKI, which is a precursor of CKD. However, the same factors, when their intensity is lower but their effects are sustained over an extended period, can also result in CKD. Heat promotes uric acid crystallization in kidney tubules, causing inflammation and damage. It triggers inflammatory responses and oxidative stress, which further harm nephrons. Intracellular calcium overload and mitochondrial dysfunction caused by heat contribute to energy deficits and tubular injury. Vascular endothelial dysfunction also occurs, leading to impaired renal circulation. Therefore, heat-related kidney diseases include AKI as an acute event, progression to CKD, and elevated risk of kidney stones from dehydration and metabolic alterations, as well as the appearance of proteinuria and hematuria [16,61].

Another important vicious circle that heat may trigger in patients with CKD is the development of electrolyte disturbances, particularly in dysnatremia. Brennan et al. found hypernatremia was 2.5 times more frequent in samples drawn during the heatwave [73]. In a cohort study involving 396 heat-exposed participants (exposure group) and 3408 unexposed participants (control group) from the general population, sodium levels were found to be statistically significantly higher in the exposed group [74].

However, in CKD, the regulatory capacity for water and sodium balance is impaired. During prolonged heatwaves, increased vasopressin secretion, sweating, altered intake, and hormonal responses may favor water retention and dilutional hyponatremia rather than hypernatremia. An international systematic review of the relationship between serum sodium levels and ambient temperature concluded that higher ambient temperatures are consistently associated with an increased incidence of hyponatremia [75]. Moreover, Mannheimer et al., using climate modelling data from Sweden, estimated that a temperature rise of just 1 °C (1.8 °F) would lead to a 6.3% increase in hospitalizations for hyponatremia [76]. Flahault et al. also analyzed whether exposure to heatwaves affects plasma sodium levels in 2964 patients with CKD stages 2–5 from the French CKD-REIN cohort. At least 3 days of heatwave within the past 8 days increased the odds of lower plasma sodium levels (including hyponatremia and mildly decreased sodium): OR 1.48 (95% CI 1.10–1.99) [77]. Similar patterns were seen for ≥4 heatwave days in the preceding 15 days [77]. These observations call into question generic public health recommendations for patients with CKD to “increase water intake” during heatwaves, because such advice may in fact heighten the risk of hyponatremia. 

Diabetes mellitus (DM), and in particular its complication diabetic nephropathy, is the leading cause of CKD around the world. Blauw et al. found that for every 1 °C increase in temperature, diabetes incidence increased by 0.314 cases per 1000 population [78]. Rising temperatures are associated with increased morbidity and mortality in individuals with DM. An Australian study found that during a moderate-intensity heat wave of 2 days, diabetes-related hospitalizations increased by 19% and deaths by 64% [79].

Diabetes disrupts the body’s ability to maintain thermal homeostasis through several pathways. Autonomic neuropathy, a frequent complication of DM, affects the nerves responsible for involuntary functions, including thermoregulation. During heat stress, individuals with diabetes exhibit decreased skin blood flow and blunted sweating responses, compromising the primary mechanisms of heat loss [80]. Under experimental conditions, Qiao et al. used congenital type 2 diabetic BKS-db/db mice and wild-type controls to test whether repeated exposure to high temperature (40 °C) and NO_2_ (5 ppm), alone or in combination, worsens diabetic nephropathy. They confirmed that both extreme heat and NO_2_, independently and synergistically, aggravate diabetic nephropathy by activating TRP channels by engaging TRP channels, intensifying inflammatory and oxidative injury [81].

Heat injury, defined as the accumulation of heat in the body beyond its tolerance limit, significantly increases the risk of developing CKD and the rate at which it progresses. Compared with individuals without heat injury, those who experienced it had an approximately 1.4-fold higher risk of reaching stage 2 CKD, a 5.3-fold higher risk of stage 3 CKD, an 8-fold higher risk of stage 4 CKD, and more than an 11-fold higher risk of stage 5 CKD [82]. This demonstrates a clear link: as CKD stage advances, the risk associated with prior heat injury exposure increases significantly. Notably, patients with heatstroke show a 4-fold increased risk of subsequent CKD and a 9-fold increased risk of progression to ESRD, requiring long-term renal replacement therapy, such as dialysis [82]. Romine et al. investigated the relationship between temperature and healthcare utilization among patients with CKD. In 916,886 CKD patients (G3–G5 stage), exposure to days with higher heat indices was associated with a statistically significant increase in weekly ED utilization, more heat-related primary diagnosis codes, and more frequent kidney-related primary diagnostic codes [83]. The DAPA-CKD trial was a global, randomized, placebo-controlled study investigating the impact of dapagliflozin on kidney and cardiovascular outcomes in patients with diabetic and non-diabetic chronic kidney disease [84]. Conducted across 393 centers in 21 countries with diverse climates, a post hoc analysis of this study revealed an association between higher ambient heat exposure and accelerated kidney function decline. Specifically, patients exposed to very hot environments experienced up to an 8% greater annual loss in estimated glomerular filtration rate (eGFR) compared to those in temperate climates, even after adjusting for seasonal variables [85].

Patients on RRT, including hemodialysis or peritoneal dialysis for over three months, represent the most severely affected subgroup with heat-related risks. Patients on dialysis are uniquely vulnerable to heat compared to the general population. Exposure to high temperature is associated with elevated risk for both mortality and health care utilization among hemodialysis patients. The first-of-its-kind study of analysed data from about 7000 Fresenius Kidney Care patients showed that on extremely hot days, same-day hospitalizations rose by 27% and same-day deaths by 31% among people with ESRD [86]. Patients with prevalent comorbidities were at an even higher risk. Mortality on the identified high-heat days increased by 83% in those with both ESRD and diabetes, and by about 60% and 55% in those living with ESRD and chronic obstructive pulmonary disease or congestive heart failure, respectively [86]. Xi et al. found that the risk ratios for all-cause mortality and daily temperature were 1.07 (95% confidence interval [CI]: 1.03–1.11), 1.17 (1.14–1.21) for fluid disorder-related hospital admissions, and 1.19 (1.16–1.22) for cardiovascular event-related emergency department visits, when comparing daily temperatures at the 99th percentile to those at the 50th percentile [87].

Emerging evidence reveals that heat poses significant risks to kidney transplant recipients, with impacts on both graft survival and long-term function. Cojuc-Konigsberg et al. presented the first results of their retrospective study that reviewed 63,351 kidney transplant recipients among 250 kidney transplant centers in the US [88]. They concluded that patients undergoing kidney transplantation in regions with the highest heat indexes show increased rates of kidney function decline and major adverse kidney events (MAKEs) compared with recipients of transplants performed in cooler environments. Increased heat exposure was associated with a progressive and faster eGFR decline. For each additional year with an average heat index of at least 30 °C (for a median follow-up period of 4.9 years), there was an average annual reduction in the eGFR of −0.62% [88]. Furthermore, kidney transplant recipients who underwent transplantation in warmer climates experienced a 30% higher risk of MAKE, which includes death from any cause, graft failure, or a doubling of serum creatinine, compared to those transplanted in cooler environments (adjusted hazard ratio 1.30; *p* < 0.001) [88].

## 3. Conclusions

As temperatures are anticipated to rise in the coming years, problems linked to heat and its harmful effects on kidney health are expected to become more frequent and severe with time. Heat stress significantly affects kidney health, causing acute and chronic kidney injury. The physiological strain caused by extreme heat exposure, through mechanisms such as dehydration, volume depletion, and increased glomerular permeability, contributes to a spectrum of kidney conditions, including AKI, nephrolithiasis, CKD, proteinuria, and hematuria. Heat waves are causing increased hospitalizations for kidney disease worldwide, with vulnerable groups, particularly outdoor workers and older adults, being at higher risk. There is an urgent need for further research to elucidate the underlying pathophysiological mechanisms of heat stress as a key factor in the etiology of kidney disease. This will help in the future development of targeted interventions, such as occupational safeguards, hydration protocols, early detection programs, and public health interventions, in order to protect at-risk groups.

## Data Availability

Data are contained within the article. The original contributions presented in this study are included in the article. Further inquiries can be directed to the corresponding author.
